# Hospital social work in acquired brain injury care: patients' and kin' experiences and perspectives on psychosocial support

**DOI:** 10.1080/17482631.2026.2654063

**Published:** 2026-04-02

**Authors:** Monika Högsnes, Katarina Grim, Camilla Udo, Evelina Landstedt, Alexandra Ullsten

**Affiliations:** a Department of Social and Psychological Studies, Karlstads University, Karlstad, Sweden; b Centre for Clinical Research and Education, Region Värmland, Karlstad, Sweden; c School of Health and Welfare, Dalarna University, Falun, Sweden; d Faculty of Medicine and Health, School of Health Sciences, Örebro University, Örebro, Sweden

**Keywords:** User-perspective, psychosocial support, acquired brain injury, healthcare social worker, next of kin

## Abstract

**Purpose:**

Acquired brain injuries (ABI) have a profound impact on both individuals and their families, making psychosocial support a crucial component of neurorehabilitation. Understanding this support is essential for enhancing the services provided by healthcare social workers (HSWs). This study aimed to explore the experiences of individuals with ABI and their next of kin regarding psychosocial support, with a particular focus on the support received from HSWs.

**Method:**

A total of 33 interviews were conducted, 23 with individuals with ABI and 10 with next of kin. Data were analyzed using qualitative content analysis.

**Results:**

Participants with ABI and their next of kin valued the support provided by HSWs in addressing challenges related to medical, emotional, financial, and social needs. The importance of informational support—such as explanations of the medical condition and treatment options—was emphasized, alongside instrumental support that helped individuals navigate complex welfare systems.

**Conclusions:**

While counselling and crisis management are essential, the informational and practical support provided by HSWs should not be regarded as secondary. The need for support varies significantly—both between individuals and over time—highlighting the importance of tailoring interventions to each person's unique circumstances in order to achieve truly person-centered care.

## Introduction

The present study focuses on a relatively under-researched area—experiences of psychosocial support provided by hospital social workers (HSWs) among individuals with acquired brain injury (ABI) and their next of kin. Consequently, the study is exploratory and relates to several research fields, for example disability, rehabilitation, informal caregiving, crisis management, and hospital social work. While a comprehensive review of all these fields would be valuable, it falls outside the scope of this article. Instead, the following outline of research provides a brief introduction to ABI and psychosocial support in the health care context, followed by a theoretical elaboration on psychosocial support.

Acquired brain injury (ABI) refers to alterations in brain function resulting from various diseases or injuries occurring during or after birth (Goldman et al., 2022). It represents one of the leading contributors to the global burden of disease (Bener et al., 2010; Tagliaferri et al., 2006; World Health Organization, 2022). Its consequences vary but often include fatigue as well as motor‑sensory, speech and language, emotional, behavioural, and cognitive impairments, which affect health-related quality of life (Åkerlund et al., 2021; Stenberg et al., 2015b; Ponsford et al., 2014). Given the life-altering impact on individuals and families following ABI, psychosocial support is a vital part of neurorehabilitation (Stiekema et al., 2020; Thøgersen et al., 2022). Health care professionals play a vital role in this regard (Buck et al., 2012; Sohlberg et al., 2024; Williams & Douglas, 2022), although no clear agreement on definitions and clinical operationalizations of psychosocial support is evident in the literature (Gehlert et al., 2019; Lombardi et al., 2024). Given this uncertainty, the expertise of HSWs is of profound importance, i.e., recognising holistic social aspects of importance for the wellbeing of vulnerable patient groups (Lombardi et al., 2024; Simpson et al., 2023). Such social orientation is exemplified by bridging health and social care, providing emotional and tangible support, as well as diagnosis‑related information to patients and their families (Gonzáles-Ramos et al., 2019; Heenan & Birrell, 2018; Moriarty et al., 2019). In Sweden, the profession “healthcare social worker” is a protected professional certification since 2019, acquired by post-graduate level education and training. The general assignment for Swedish HSWs is to support and advocate for the psychosocial perspective for both inpatients and outpatients, including specialised and tailored psychosocial support to strengthen the coping capacities of patients and their families (NBHW [National Board of Health & Welfare], 2014). This includes, for example counselling, care planning, diagnosis-related information, psychoeducation, and engaging in contacts with authorities and case management (Lilliehorn et al., 2019; Sjöström, 2013).

While research findings are inconsistent as to whether HSW psychosocial support is effective for individuals with ABI (Matérne et al., 2022b; Stiekema et al., 2020), several studies suggest that HSW psychosocial support helps individuals manage crises and reduces the risks of depression, anxiety, and suicide (Glintborg & Hansen, 2021; Madsen et al., 2018; Rossom et al., 2023; Stiekema et al., 2020). Patient experiences highlight the importance of psychosocial support in fostering security, predictability, and hope both immediately and long after the injury (Abrahamson et al., 2017; Glintborg & Hansen, 2021; Matérne et al., 2022b). For the ABI group, research indicates a positive role of HSW in understanding ABI (Holloway & Tasker, 2019; Mantell et al., 2017; Linden et al., 2023; Norman et al., 2020) and accommodating the needs for coordinated support involving families and professionals (Holloway & Norman, 2022; Holloway & Tasker, 2019; Lannin et al., 2014; Leonard et al., 2024; Stiekema et al., 2020).

Despite insights from existing research on the importance of including user perspectives in knowledge production and the development of high-quality services (Grim et al., 2022), previous research in the field of hospital social work has primarily focused on the views of health professionals (Sernbo, 2019). Applying a user-centred approach is highly relevant in general to advancing knowledge within the field and improving professional practice. This is particularly important for individuals with ABI and their next of kin, as research indicates that psychosocial support often does not meet their needs (Holloway & Tasker, 2019; Lehnerer et al., 2019; Norman et al., 2020; Piccena et al., 2016). For example, studies on stroke and traumatic injury survivors show that both patients and their next of kin report difficulties navigating healthcare systems (Matérne et al., 2022a; Stenberg et al., 2022a) and that next of kin are often unprepared for the sudden and significant role changes that follow a stroke (Camak, 2015; Intamas et al., 2021; Tziaka et al., 2024). The introduction of a formal professional HSW certification has further highlighted the need for evidence-based person-centred social work practice in healthcare. Advancing the knowledge of psychosocial support from a user perspective, both affected individuals and their families, is an essential part of this development (Grim et al., 2022; McCance & McCormack, 2023; Sernbo, 2019; Udo et al., 2019).

As noted above, psychosocial work in healthcare, including psychosocial interventions such as provision of psychosocial support, draws on general social work theory which emphasises how individual experiences of health, illness and rehabilitation are shaped by social context (Gehlert et al., 2019; Mannsåker & Vågan, 2024; Ståhl & Lundälv, 2023). However, specifications tend to describe practice rather than conceptual or theoretical elaborations, for example strengthening individuals' ability to cope with internal and external stressors, guiding and orienting them in society's various institutions and regulations, and working for changes in the social environment so that people's life situations develop favourably (Lilliehorn et al., 2019; Mannsåker & Vågan, 2024). In the absence of a clearly outlined theoretical grounding of psychosocial support in a healthcare context, and in keeping with a distinct social focus, we turned to Langford et al. (1997) conceptual model of social support, which categorises support into emotional, instrumental, informational, and appraisal types (Langford et al., 1997). According to Langford et al. (1997), emotional support includes providing care, empathy, love, and trust, while informational support involves offering advice and guidance to help individuals make decisions or solve problems. Instrumental support refers to providing tangible and practical assistance, and appraisal support provides useful information for self-evaluation, including affirmation, feedback, and social comparison (Langford et al., 1997). While Langford and colleagues describe social support in a broad sense—including support derived from family and friends—these conceptualisations have recently been adapted and applied in studies examining support provided by healthcare professionals (Bjørlykhaug et al., 2021; He et al., 2025; Stanikic et al., 2024). They were therefore considered relevant to apply in the present study.

Focusing on support received from hospital social workers, the aim of this study was to explore and describe the experiences and perspectives of psychosocial support received in healthcare contexts among individuals with ABI and their next of kin. This includes distinguishing various aspects of support, as well as identifying both met and unmet support needs.

## Materials and methods

### Design

This explorative qualitative study builds on semi-structured interviews with persons with ABI and with next of kin. We use an abductive approach where the inductive, descriptive approach predominates and where theoretical concepts are applied in the discussion section when the inductively derived findings are reflected upon. The research process and reporting adhered to the Consolidated Criteria for Reporting Qualitative Research (COREQ) checklist (Tong et al., 2007).

### Sampling and study participants

A total of 33 individuals participated in the study, of whom 23 had ABI and 10 were next of kin. Participants were recruited through a snowball sampling strategy by announcing information about the study via patient organisations, healthcare units, and social media. Inclusion criteria were adults (>18 years old) with ABI or next of kin who had met with an HSW at least twice in connection with the ABI diagnosis. Persons with cognitive and verbal capabilities to take part in an interview were included. Participants were exclusively recruited from other regions than where the first author is professionally active as an HSW.

Four out of five contacted patient organisations agreed to share information about the study with their members. This invitation was also distributed via various social media platforms, including Facebook, LinkedIn, and Instagram. The first author contacted unit managers at thirteen different ABI healthcare units across Sweden via email or regular mail, requesting their assistance in sharing information about the study within their units and with their HSWs. These units were located in seven different Swedish regions, selected to ensure geographical diversity and variation in diagnostic focus, including rehabilitation, habilitation, and neurological care. Responses varied: some units did not reply, others declined to participate, one agreed to distribute the written information internally, and another initiated direct contact with the first author to learn more. This unit also asked individuals within their organisation if they were interested in participating and granted permission for professionals to share contact details with the first author.

Interested individuals expressed their willingness to participate through a digital link, by telephone, or via a representative from a patient organisation or healthcare unit. The first author contacted each respondent to verify eligibility and provide further details about the study. Two individuals did not meet the inclusion criteria, and three others later withdrew due to personal reasons.

Eligible participants received an information letter and consent form, and interview appointments were scheduled accordingly.

As shown in Table I, participants were recruited from ten counties across Sweden, representing both cities and smaller municipalities. The causes of ABI included traumatic incidents such as traffic accidents and falls, as well as medical conditions such as tick-borne encephalitis and stroke. The time since injury ranged from one to forty years. Among the next of kin who participated, relationships included either parents or spouses.

**Table I.** Characteristics of participants and interview procedures.

Participants (*N* = 33)	Individuals with acquired brain injury (*n* = 23)	Individuals who were next of kin (*n* = 10)
Gender		
* Female*	12	7
* Men*	11	3
Age		
* 22–30*	2	0
* 31–42*	3	0
* 43–55*	8	1
* 55+*	10	9
Place of residence		
* Big city*	10	3
* Small town*	13	7
Type of acquired brain injury		
* Traumatic brain injury*	11	3
* Stroke*	10	6
* Other illness affecting the brain*	2	1
Interview procedures		
* Individual interview*	18	3
* Joint interview: person with acquired brain injury and their next of kin*	5	5
* Joint interview: Spouses, parents of an adult with an acquired brain injury*	0	2
Interview mode		
* Zoom*	9	5
* Telephone*	0	1
* In person*	14	4

### Data collection

The interviews were conducted between November 2023 and September 2024 by the first author. Interviews were conducted either face-to-face or digitally, depending on participant preference. In-person interviews (*n* = 18) took place in various locations, including participants' homes, patient organisation offices, healthcare unit offices, libraries, or cafés. Digital interviews were conducted via Zoom (*n* = 14), and one interview was held by phone. Five participants with ABI chose to be interviewed together with a next of kin (parent or spouse), and two next of kin (spouses) chose to be interviewed together. These individuals expressed that participating in the interview together would facilitate communication and the recall of earlier experiences. Of the ABI-affected individual and next of kin pairs, three were adult children with ABI with parent and two pairs were ABI-affected individuals with a spouse. The remaining interviews were conducted individually.

The interviews were semi-structured, guided by an interview protocol consisting of open-ended questions about experiences and views on initial and subsequent meetings with HSWs, types of psychosocial support received, what forms of support were perceived as helpful or unhelpful, participants' impressions of the HSWs' approach and knowledge about ABI, and concrete examples from their encounters. The interview guides were similarly worded for individuals with ABI and for next of kin. The preliminary interview guide was developed based on the study's aims and a review of relevant literature. It was refined through discussions within the research team. The first participant was invited to provide feedback on the guide and agreed to do so. As a result, the interview questions were arranged in chronological order.

Interviews lasted between 45 and 90 minutes and were digitally recorded. Transcripts were anonymized and coded in such a way that individual statements could not be traced back to specific participants.

### Ethical considerations

Participants gave written informed consent after receiving both verbal and written information about the study, and were reminded of their right to withdraw at any time. Before the interviews, they were informed that they could pause if needed, though no one requested a break. Psychosocial support in the form of a counselling session was also offered post-interview, but none of the participants utilised it. The study was approved by the Swedish Ethical Review Authority (Dnr 2023-03387-01; Dnr 2024-01920-02), in accordance with national legislation.

### Data analysis

The digital audio files were verbatim transcribed via the voice-to-text software service Amberscript. The first author (MH) listened to all interviews, reviewed the transcripts and corrected errors. Transcripts were transferred to NVivo 14 software for further data management and analysis.

Qualitative content analysis was applied (Graneheim et al., 2017). This method was deemed appropriate for achieving the aim of capturing both similarities and differences in the wide variety of experiences (Graneheim et al., 2017). The analysis began with a repeated review of the data to gain a sense of the whole. In the next step, the data were read word by word, and meaning units—i.e., words or phrases connected by content or context—were highlighted. These meaning units were then condensed to make the text shorter and more manageable, while preserving the central content and ensuring that nothing essential was lost. Next, the condensed text was assigned codes that captured key thoughts or concepts related to the aim of the study. In the following abstraction process, these codes were categorised into preliminary categories based on their relationships and connections (Graneheim et al., 2017). This step of the analysis process was undertaken by the first and last authors. Next, the middle authors reviewed the preliminary categorisations to provide analyst triangulation (Malterud, 2001). In the final stage of analysis, all authors were involved in refining the final categories and subcategories.

Table II presents an example of the qualitative content analysis process. During the analysis, it became evident that Langford et al. (1997) theoretical framework of social support could add depth to the understanding of participants' accounts. Consequently, after describing the categories and subcategories in the results section—providing a primarily descriptive account—we applied Langford et al. (1997) framework to discuss the findings in terms of *instrumental, informational, emotional,* and *appraisal support,* and in light of other previous literature.

**Table II.** Illustration of the qualitative content analysis process.

Meaning unit	Condensed meaning unit	Code	Sub-category	Category
I've received help with economic papers, insurances and stuff like that	The hospital social worker helped with economic paper work	Hands-on support in financial issues	Support in managing practicalities	Navigating and advocating in complex health systems

#### Authors' preunderstanding

The authors' preunderstanding and its potential influence on the analysis and interpretation of psychosocial support and ABI have been continuously reflected upon and discussed throughout the different phases of the study (Alvesson & Sandberg, 2021). MH and CU have clinical preunderstanding as healthcare social workers, while AU has clinical experience in brain injury rehabilitation as a music therapist. MH also has long clinical experience within neurorehabilitation. As for academic specialisation, the authors contribute with knowledge perspectives from the fields of social work (MH, CU, EL, KG), public health science (EL), and care science (KG, AU).

A reflexive approach was embedded throughout the research process, with the researcher actively acknowledging their positionality. In particular, the authors with clinical experience related to the support examined in the study were especially mindful of the need to critically reflect on personal biases and assumptions that could influence the interpretation of the data. Open and continuous dialogue at all stages of the analysis process was essential to prevent individual preconceptions from unduly influencing interpretations. All authors participated in the analysis to provide multiple perspectives, ensure consensus, and strengthen the trustworthiness of the findings. These discussions enabled the team to jointly identify nuances and patterns in the data that might not have been recognised individually (Alvesson & Sandberg, 2021).

## Results

Table III shows an overview of the categories and sub-categories. Since we had no intention of comparing and highlighting differences and potential conflicts in the perspectives of different stakeholders, the perspectives of ABI-affected individuals and their next of kin were jointly analysed and presented in a predominantly comprehensive analysis. Where deemed possible and relevant, these two positions are clarified, and the role of the respondent is consistently indicated in the quotations. Quotations from participants with ABI are referred to as A1–23, and those who are next of kin as B1–10.

**Table III.** Categories and sub-categories reflecting experiences and perspectives of individuals with acquired brain injury and next of kin regarding psychosocial support provided by hospital social workers.

Categories	Sub-categories
Competencies needed for establishing trust	Being knowledgeable about acquired brain injury and its consequences Having the ability to build interpersonal chemistry
Support in coping with altered conditions	Crisis management Relational support
Navigating and advocating in complex healthcare systems	Support in managing practicalities Managing collaboration with multiple actors

### Competencies needed for establishing trust

The first category concerns the characteristics of HSWs that were highlighted as important for establishing trust. This involves knowledge about ABI and the needs of those affected and their next of kin, as well as more intangible, soft skills that are needed to create good rapport and an alliance. Consequently, this category consists of two subcategories: *Being knowledgeable about ABI and its consequences* and *Having the ability to build interpersonal chemistry.*

#### Being knowledgeable about ABI and its consequences

The participants emphasised the role of interpersonal trust in determining the quality and relevance of the received support. A key factor in this process was the HSW's knowledge and ability to communicate about their or their next of kin's condition and how it may affect their lives. One participant with ABI described the relief he felt when an HSW explained what mental fatigue was, as it supported his understanding of himself. The father of a son with ABI expressed how a HSW's knowledge about ABI and its consequences was essential for adapting communication appropriately and asking his son the right questions: “I felt that she (the HSW) had the tools to have a conversation with my son, she had the knowledge to ask questions that fit him at the level he needs and, above all, not to put him down.” (B–10).

On the contrary, when HSWs had poor knowledge about the disability or lacked essential communication skills, the interaction between participants and the HSW was negatively affected, leading to frustration, worries, and reduced hope for the future. In the following quote, a woman with a traumatic brain injury describes her frustration at encountering a HSW that lacked diagnosis-relevant knowledge; “Then I met another HSW, but I couldn't bear to hear her saying, “I don't understand this. I don't know anything about what you're saying.” I couldn't talk to her. I just couldn't cope with it.” (A–3).

Others experienced situations where the HSWs failed to provide sufficient answers and instead redirected questions back to the participants themselves.

#### Having the ability to build interpersonal chemistry

This category concerns alliance building and the importance of interpersonal chemistry between HSWs and the affected person or their next of kin. In the following quote, a man who had suffered from a stroke describes how meeting a HSW with whom he had good chemistry gave him confidence that HSW would do their utmost to help him:

*When it comes to contact with HSWs and people in healthcare in general, I think interpersonal chemistry is very important. It's important to feel that you can put your hands in someone's hands and feel safe with this person. I know that the HSW is trying to do her best based on her circumstances. Knowing that she is a good person makes me feel safe. It is enough for me to trust her.* (A–13)

To experience this sense of interpersonal chemistry, it was essential for the participants to be seen as individuals in a holistic sense. From the participants' descriptions, it seemed that they had a clear perception of whether the HSW had a holistic view of them or not. For them, a holistic view became apparent when an HSW addressed them by their name during the inpatient phase, asked questions related to their whole life situation, and showed that they understood their needs and could support them with both practical and emotional issues. Participants in both inpatient and outpatient settings further expressed appreciation for being validated as a person “beyond” the condition, by, for example, chatting about similar music interests or what life was like before the injury or illness. These sentiments are reflected in the following quote by a woman who had suffered from a stroke; “It was great that she (HSW) could see me as an individual, that I was not just someone with a stroke, that I was [name]. That was really important to me and it felt very good.” (A–7).

However, identifying what contributed to experiences of interpersonal chemistry was also less obvious. Some participants perceived it as an instant feeling or as a sense of safety in the room, while others described it as a process over time. Defining the absence of interpersonal chemistry with the HSW could be equally challenging, as reflected in the account of one participant, a woman who had suffered a stroke:

*I didn't think it was unpleasant meeting the HSW, but it's like that it hasn't really clicked between us… I felt that I did not really get a connection with her… Or that she did not show a real understanding or experience of my situation.* (A–15)

Overall, the accounts of both persons with ABI and their next of kin reflect that key attributes for creating interpersonal chemistry were the ability to listen closely, show patience, ask questions, and display a strong commitment and genuine interest in and care for the person with ABI and their families.

### Support in coping with altered conditions

The second category relates to the process of adapting to the new circumstances resulting from injury, illness, and disability. Both individuals with ABI and their next of kin expressed a need for support in coping with thoughts related to grief, death, suicide, and survival. They also needed guidance in coming to terms with the new reality and understanding its impact on everyday life. The category comprises two sub-categories: *Crisis management* and *Relational support*.

#### Crisis management

Participants with ABI and their next of kin commonly described experiences of trauma or crisis related to the injury or illness. They emphasised the importance of having the opportunity to process these traumatic experiences in relation to the event. Those who had received adequate crisis support from a HSW during the acute phase ascribed it profound importance. This early support contributed to a sense of safety and instilled hope for the future at a time when they felt most vulnerable, as noted by one participant, a man who had suffered from a stroke:

*The HSW was there for me as a very safe haven when we met… It wasn't about long lectures, just three simple things—maybe* *“Walk today, move around, write a diary”—so I could get out what I was feeling. He kept reminding me, “It's a crisis now, it's hard, but it will get better.” He became a source of hope and safety for me, and he was very steady in how he treated me.* (A–6)

This particular ability to provide hope was described by some participants as a crucial element of the support they received from their HSWs. Those with less positive experiences in this regard expressed strong negative emotions and a deep mistrust towards the support they had received. In some cases, this led participants to reject further assistance from HSWs altogether. Several participants expressed anxiety when meeting a new HSW, fearing that the experience would once again diminish their sense of hope. One next of kin participant (a mother of a daughter who had a severe stroke), emphasised the importance of HSWs conveying hope and shared her frustration when this essential aspect of support was lacking:

*I felt that the most important thing she (the HSW) could have been was a bearer of my hope… But I think that most HSWs I've met tend to ask questions as if they expect me to have the answers—as if I'm the one who should figure it all out. But that's not what I need in those moments. I need good examples. Like,* *“Think about how it could turn out differently. Think about how many times you've worried in life, and it never turned out as badly as you feared.” That's what I needed—those kinds of reminders. Not doomsday prophecies.* (B–3)

Participants described facing significant challenges in adapting to their new circumstances, often due to the uncertainty surrounding the permanence of their difficulties. Many emphasised the importance of learning strategies to cope with their new reality. However, HSW advice on coping strategies needed to be appropriately timed. When individuals with ABI or their next of kin were in a phase where they primarily needed to express their emotions and share their experiences, a focus on strategies could feel premature or even dismissive. The following quote from a woman who had had a stroke illustrates the frustration that can arise when a HSW emphasises strategy over emotional support:

*I wanted to talk to my HSW about why I feel like this after my stroke. I was getting homework—sure, that's fine—* *“do this, think like this.” But that wasn't what I needed. I needed to be seen, and to have someone I could talk to.* (A–7)

While many participants described positive experiences with trauma and crisis support, there were also some accounts of such support being insufficient or entirely lacking. On the other hand, some participants shared that they were not initially receptive to support from the HSW during the early stages of trauma or crisis. However, they emphasised that it was still important for the support to be offered. Even if they would have declined help at the time, they expressed that simply being asked would have held value. Knowing that support was available would have made it easier to reach out later, when needs arose.

#### Relational support

The accounts of next of kin participants clearly revealed struggles with sorrow, grief, and the challenges of adjusting to a new everyday life, particularly changes in close relationships. Participants described how these relationships often changed dramatically—sometimes overnight—for both the individual with ABI and their next of kin. Participants emphasised the importance of ensuring that next of kin, including both children and adults, were also offered support. They also stressed that such support should be extended to *all* next of kin, not just a select few. Many participants noted that access to caregiver support often felt arbitrary, with some receiving it while others were never offered any assistance at all.

Other central components of psychosocial support needs expressed primarily by participants with ABI were romantic relationships and intimacy, often overlooked yet deeply important aspects of life. They acknowledged that these topics can be sensitive and shared that they often experienced difficulties in speaking openly about deeper emotions with their HSWs. A man who had suffered a stroke and who was single shared his thoughts; “I've missed talking with my HSW about love relations, because it's been difficult for me, how to live together and to be able to have a partner.” (A–15).

Close relationships emerged as a particularly important area of psychosocial support needs. Participants not only expressed regret over not having initiated conversations about these issues with their HSW, but also highlighted a perceived silence or lack of initiative from the professionals in addressing these sensitive topics.

### Navigating and advocating in complex healthcare systems

The third category involves participants' reflections on support and guidance from HSWs in managing administrative tasks around healthcare, sick leave, insurance issues, and social support that arise in their new situation, in ensuring that one's needs and rights are met and in coordinating contacts between multiple agencies. Accordingly, the category consists of two subcategories: *Support in managing practicalities* and *Managing collaboration with multiple actors.*

#### Support in managing practicalities

The participants - both individuals with acquired brain injury (ABI) and their next of kin - perceived that the Swedish healthcare and support systems are particularly complex to navigate. This was described as particularly challenging in times of crisis and in the presence of milder cognitive impairments resulting from ABI, for example mental fatigue. Consequently, participants from both groups ascribed immense importance to the provision of information on common practical matters, such as available welfare services and various types of support as well as HSW-led hands-on assistance including completing forms and communicating with different welfare services. A mother of a son who had suffered from a stroke described in the following quote her need for concrete support in what to do when a family member was injured:

*HSWs need to share a to-do-checklist with us after an incident like this… You are so shocked by what happened to your loved one, and you have so many practical things to do when you have to go to the hospital…You need all the support you can get. Even with the practicalities as well.* (B–8)

Participants expressed a need for HSWs to act as advocates on their behalf to help them access the support they may be entitled to. This need was particularly evident in relation to navigating social legislation and dealing with institutions such as the Social Insurance Agency, Employment Services, and Social Services. It also included support with matters like insurance, financial assistance, transport services, dental care cards, and attestations for extended preschool hours. A woman who had suffered from a stroke and was struggling with fatigue as a consequence of the stroke described it in this way; “I get tired easily…so it means everything to me actually, to receive help with paperwork and guidance in how healthcare and social insurance work.” (A–17).

Both individuals with ABI and their next of kin described this advocacy support as highly important in reducing their stress. They found it positive to be asked whether they needed practical or advocacy support, even if they did not have such needs at the time. Several next of kin expressed that during an acute crisis, they did not have the capability to engage with an HSW on these matters. However, they appreciated knowing where to turn for help once they were ready. Having that information in advance was helpful when the time came.

#### Managing collaboration with multiple actors

Receiving support from HSWs in coordinating professionals from multiple services and agencies was highly valued by the participants. Such coordination was closely linked to whether the HSW adopted what they perceived as a holistic approach. This involved the HSW being proactive in identifying a wide range of needs, not only focusing on counselling related to the emotional and psychological impact of illness or injury, but also on connecting them with social services essential for managing everyday life.

When discussing the HSW's role in managing collaboration between multiple actors, participants highlighted both the importance of inviting relevant parties to meetings, and the HSW's active involvement in reaching out to various stakeholders. This included informing others about the needs of the patient and their family, as well as collecting necessary information. Moreover, participants expressed expectations that the HSW would take an active role during meetings and thereby represent the patient and their next of kin to ensure their voices were heard. While several positive experiences were revealed, there were instances when the HSW either did not attend meetings with other parties - such as hospital discharge meetings - or attended without taking an active role. As described by a woman who had had a stroke:

*During a meeting with other professionals, I thought the HSW would be supportive and say,” This is important for her if she is going to be involved in a work placement and she has these challenges due to her ABI…” But the HSW said nothing, and I had relied on the HSW to speak for me. I was very disappointed in how HSW acted and wished I had prepared myself better for the meeting.* (A–5)

These experiences underscore the critical role of HSWs in facilitating effective interprofessional collaboration, and how proactive engagement and advocacy by the HSW were essential for ensuring that participants' needs and perspectives were adequately represented.

## Discussion

The present study examined psychosocial support from the users' perspective, both patients and their families, providing additional insight into the concept of psychosocial support in the context of hospital social work. Key findings highlight that, although counselling and crisis management are important, the informational and practical support provided by HSWs should not be considered secondary. Psychosocial support ought to be offered early following ABI in order to prevent further suffering. The need for support varies both between individuals and over time, underscoring the importance of listening and tailoring the support to each person's unique needs to achieve person-centred care.

In line with previous research (Gonzáles-Ramos et al., 2019; Holloway & Tasker, 2019; Mantell et al., 2017; Linden et al., 2023; Norman et al., 2020), persons with ABI and their next of kin experienced diagnosis-relevant knowledge and understanding of social legislation services connected with ABI care and rehabilitation as essential dimensions of psychosocial support provided by HSWs. Clearly, those HSW who met the formally expected capacities of navigating and linking biomedical, psychological, and social perspectives in their professional practice (Lilliehorn et al., 2019; Ståhl & Lundälv, 2023) were perceived positively in terms of providing adequate support. This process of trust building through expertise and authentic engagement relates to informational support (Langford et al., 1997) - HSWs who provided thorough and relevant informational support were perceived as more trustworthy, helpful, and efficient in providing psychosocial support.

Providing informational support such as facts, advice, or guidance, also appears to be a prerequisite for adequate *emotional support,* including empathy, validation, and encouragement (Langford et al., 1997). In accordance with previous literature (Sohlberg et al., 2024; Thøgersen et al., 2022; Williams & Douglas, 2022), persons with ABI and their next of kin perceived *informal and emotional support* as fostering interpersonal chemistry—a central component in the formation of trusting relationships. The findings highlight the importance of the HSW showing genuine interest, being deeply engaged, and maintaining a holistic view of them and their situation. Again, authentic engagement by the HSW, for example not only listening attentively but also asking relevant questions, was perceived as crucial. Clearly, these highly empathetic qualities are prerequisites for providing *emotional support*, one of the most widely discussed aspects of social support (Langford et al., 1997). In agreement, this analysis demonstrates how the lack of informal and emotional dimensions of psychosocial support exemplifies unmet needs, findings supported in previous research (Holloway & Tasker, 2019; Lehnerer et al., 2019; Matérne et al., 2022a; Norman et al., 2023; Stenberg et al., 2022a). This study exemplifies that an insufficient ability to identify the specific information and communication needs of individuals with ABI or their next of kin may result in a failure to establish solid connections with individuals in crisis. Poor knowledge regarding social service processes, crisis management, and condition-related issues such as ABI and mental fatigue may undermine trust in the HSW and impair interpersonal chemistry.

Participants' accounts also relate to the importance of social embeddedness - the way individuals connect with other people in their social environment—and reciprocal appraisal support as key foundations for beneficial psychosocial support. According to Langford et al. (1997), central mechanisms of beneficial appraisal support involve receiving feedback and information that helps someone evaluate their own feelings, behaviours, or situations, which can aid in self-assessment and understanding. The present study highlights the particular importance of HSWs' ability to establish a sense of security and hope. In so doing, the HSW creates a social climate where psychosocial support can take place (Abrahamson et al., 2017; Glintborg & Hansen, 2021; Matérne et al., 2022b).

In accordance with previous findings (Holloway & Norman, 2022; Holloway & Tasker, 2019; Lannin et al., 2014; Leonard et al., 2024; Stenberg et al., 2022a; Stiekema et al., 2020), our analysis highlighted the importance of HSWs working cooperatively with other professionals and with family members. The need for such *instrumental support* from the HSW included hands-on assistance, such as help in filling out forms in ABI-related social legislation issues and processes, and in coordinating social and professional networks around the individual. HSWs with a strong guiding presence were highly valued, helping individuals to express themselves in meetings with other professionals within the rehabilitation system, as well as advocating on their behalf in such interactions. According to Langford et al. (1997), *instrumental support* is tangible assistance, a form of support that the present study confirms as a key source of safety and anchorage in times of crisis, when relying on a range of welfare services and thus having to navigate complex welfare systems. Providing advocacy and tangible support in service navigation emerges as a critical function of HSWs, reinforcing their role as anchors in the lives of individuals facing crisis and uncertainty.

Participants in the study described facing a range of problems, including coming to terms with their new condition, processing traumatic experiences related to ABI, coping with grief, sorrow, crisis, and thoughts of death, and navigating changes in personal relationships. Consistent with results from other studies (Glintborg & Hansen, 2021; Lannin et al., 2014; Leonard et al., 2024; Madsen et al., 2018; Rossom et al, 2020; Lilliehorn et al., 2019; Stiekema et al., 2020), the HSW was perceived as an important resource in helping them to develop strategies to handle their new situation in life. Supporting and guiding individuals in this way may be conceptualised as a form of evaluative social support, or *appraisal social support*, referring to interventions with psychoeducational and self-evaluative elements (Langford et al., 1997).

Consistent with earlier findings, support needs emerged not only in the immediate aftermath of the ABI but also continued to be significant over time (Abrahamson et al., 2017; Matérne et al., 2022b). Accordingly, the analysis highlights the critical role of the HSW in providing psychosocial support during the acute phase following an ABI—not only to individuals with ABI but also to their next of kin—thereby fostering a sense of security, predictability, and hope (Matérne et al., 2022b). An awareness and proactivity among HSWs in ABI care are essential for timely informing patients and their next of kin and address their potential psychosocial needs throughout the lifespan. In other words, HSW-led psychosocial support is not static—it represents dynamic processes through different phases relating to interpersonal relationships and time: from crisis to continuity - supporting people now and into the future.

### Methodological considerations

In keeping with the principles of trustworthiness in qualitative research, we employed several strategies to enhance credibility, dependability, and confirmability (Lindgren et al., 2020) and wish to address key aspects of those. The reflexive and transparent approach (Alvesson & Sandberg, 2021) in the multidisciplinary research team throughout all stages of the study, strengthened the analysis and the study's trustworthiness. Employing analyst triangulation strengthened the credibility of the findings (Malterud, 2001).

The decision to allow participants to be interviewed together, and to conduct a joint analysis of the experiences of people with ABI and their next of kin, can be seen as both a limitation and a strength. These two groups have different perspectives that may be contrasting. However, for our study, we chose to give respondents the opportunity to be interviewed together. This approach enabled those with greater cognitive or communicative challenges to take part, supported by their next of kin who could assist with memory, expression, and emotional comfort. This gave us the opportunity to capture a broad spectrum of experiences, allowing the voices of those with more extensive impairments to be represented. When presenting quotes, distinctions were made between the two groups to highlight differences in perspectives. Specifying each participant with a number in the quotations contributes to the credibility of the results as it demonstrates that the data are reported from a broad range of respondents (Graneheim et al., 2017).

The data for this study were collected in Sweden, where the healthcare system has several distinctive features that may limit the transferability of the findings to other healthcare systems. However, the role of HSWs is similar across various national contexts (Gonzáles-Ramos et al., 2019; Lehnerer et al., 2019; Norman et al., 2020; Piccena et al., 2016), suggesting a broader relevance to other countries' healthcare systems.

### Implications for practice

The current study highlights the breadth and complexity of the various aspects of social support that people with ABI and their next of kin need from healthcare social workers. It emphasises the importance of healthcare social workers having a broad range of knowledge to pass on to those affected, and to practice with humanity. This includes being able to perform a psychoeducational function in terms of diagnosis-relevant knowledge, knowledge of crisis management and strategies for coping with a new life situation, as well as knowledge of various welfare services and authorities and of relevant legislation. While counselling is often emphasised as the primary role of the HSW, especially in the Swedish context (Sjöström, 2013), the current study underscores the critical importance of other responsibilities that should not be overlooked in comparison. These include service navigation—connecting patients and families with community resources such as financial assistance and residential support—as well as advocating for patients' rights and needs within the welfare system. Their function as a link between health and social care, as well as between healthcare and the community was underscored. Including and offering support to next of kin emerged as a central component of the support provided. This was emphasised by both next of kin and those affected by ABI. The current study highlights a need for enhanced HSW training in ABI-related knowledge. By capturing a user perspective on the support received and the support that is perceived to be lacking, our findings clarify how support needs vary, both from person to person and during the different phases that those affected go through. This underlines the importance of consistently listening to and responding to each individual's needs in order to achieve person-centred care and support.

## Data Availability

The dataset is not publicly available due to Swedish Publicity and Confidentiality Act but are available from the corresponding author on reasonable request. Ethical approval for the study was obtained from the Swedish Ethical Review Authority (Dnr 2023-03387-01; Dnr 2024-01920-02), based on national legislation. Any further underlying data will be made available upon reasonable request.
